# Evolving molecular classification of aggressive B‐cell lymphoma

**DOI:** 10.1111/his.15350

**Published:** 2024-11-15

**Authors:** Stefan K Alig, Björn Chapuy, Daisuke Ennishi, Kieron Dunleavy, Daniel J Hodson

**Affiliations:** ^1^ Department of Internal Medicine III Ludwig Maximilian University (LMU) Hospital Munich Germany; ^2^ Department of Hematology, Oncology and Cancer Immunology Charité‐University Medical Center Berlin Berlin Germany; ^3^ Center for Comprehensive Genomic Medicine Okayama University Hospital Okayama Japan; ^4^ Department of Hematology Lombardi Comprehensive Cancer Center Washington DC USA; ^5^ Cambridge Stem Cell Institute and Department of Haematology University of Cambridge Cambridge UK

## Abstract

This review aims to provide an overview of the latest developments in the classification and molecular understanding of aggressive B‐cell lymphomas, specifically focusing on diffuse large B‐cell lymphoma (DLBCL) and high‐grade B‐cell lymphoma (HGBL). Advances in molecular techniques have led to novel ways to classify these lymphomas based on clinical, histological, transcriptional, and genetic properties. While these methods have predominantly focused on the malignant compartment, recent studies emphasize the value of profiling the tumour microenvironment for a more comprehensive disease classification. Additionally, the integration of liquid biopsies represents a promising advancement, offering less invasive and dynamic insights into tumour characteristics and treatment response. Although molecular profiles are not yet routinely used to guide therapy, emerging data highlight their potential to predict responses to novel treatments. It is our belief that integrating molecular profiling and liquid biopsies into clinical practice and research now will pave the way for more personalized and effective therapies in the future.

## Graphical abstract

Aggressive lymphomas represent a spectrum of disease entities, defined using clinical, histological, and molecular features. Gene expression and genetic profiling data add further granularity, but consensus is lacking on how these should be applied. We discuss a pragmatic approach to the evolving molecular classification in clinical trials and routine practice.

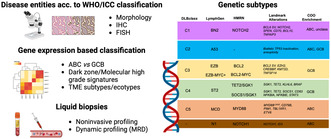

**Abbreviations**
ABC: Activated B‐cell
CAR‐T: Chimeric Antigen Receptor T‐cell
CNS: Central Nervous System
COO: Cell of Origin
ctDNA: Circulating Tumor DNA
DHIT: Double Hit Lymphoma
DHITsig: Double Hit Signature
DLBCL: Diffuse Large B‐Cell Lymphoma
DZ: Dark Zone
FFPE: Formalin‐Fixed Paraffin‐Embedded
FISH: Fluorescence In Situ Hybridization
GCB: Germinal Center B‐cell
GEP: Gene Expression Profiling
HGBL: High‐Grade B‐cell Lymphoma
ICC: International Consensus Classification
IHC: Immunohistochemistry
IPI: International Prognostic Index
LZ: Light Zone
MGH: Molecular High Grade
MRD: Minimal Residual Disease
NOS: Not Otherwise Specified
OS: Overall Survival
PCNSL: Primary Central Nervous System Lymphoma
PFS: Progression‐Free Survival
R‐CHOP: Rituximab, Cyclophosphamide, Doxorubicin, Vincristine, and Prednisone (chemotherapy regimen)
RNA‐Seq: RNA Sequencing
TME: Tumor Microenvironment
ViPOR: Venetoclax, Ibrutinib, Prednisone, Obinutuzumab, and Lenalidomide (combination therapy)
WHO: World Health Organization
WHO‐HAEM5: 5th Edition of the WHO Classification of Hematolymphoid Tumors

## Current classification systems of aggressive lymphoma

**Figure 1:**
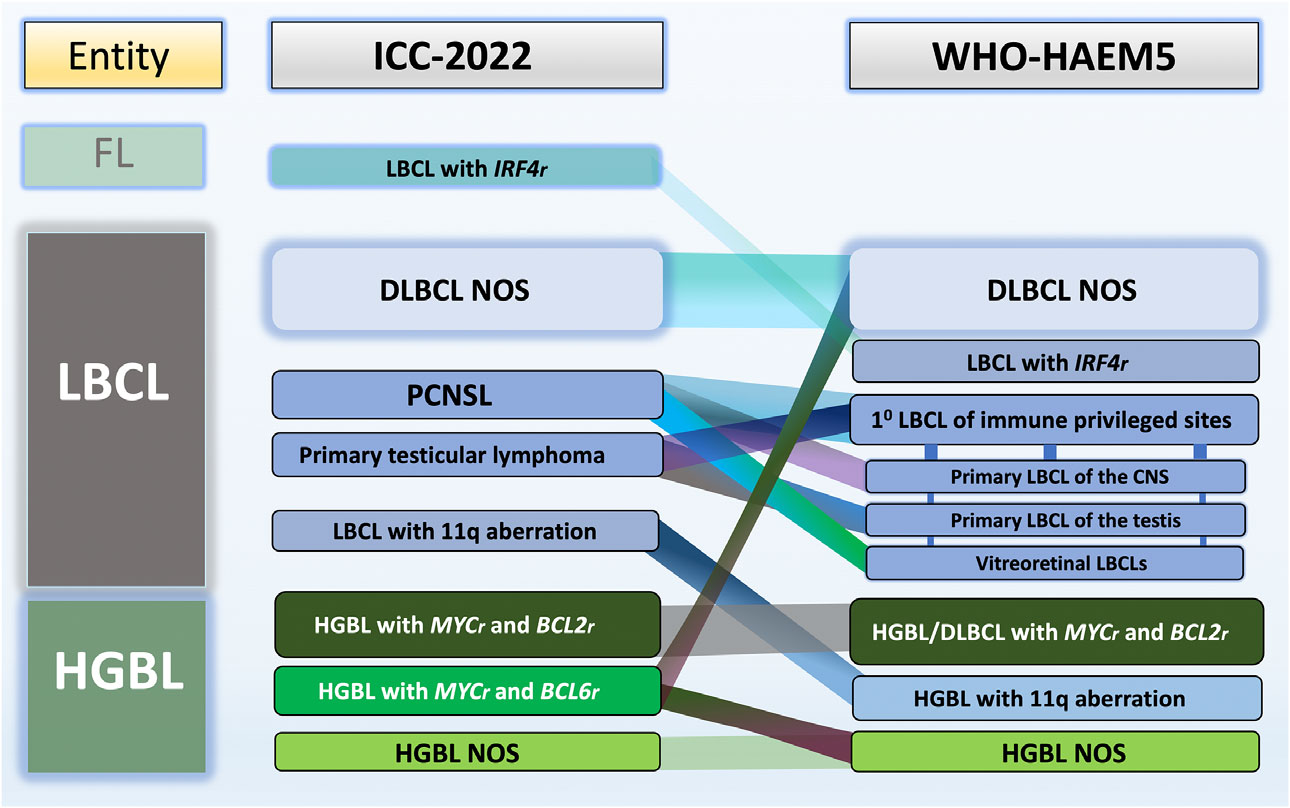
Updates on the main differences between LBCL and HGBL from ICC‐2022 and WHO‐HAEM5 Classifications.^4^ DLBCL, diffuse large B‐cell lymphoma; FL, follicular lymphoma; HGBL, high‐grade B‐cell lymphoma; ICC, International Consensus Classification; LBCL, large B‐cell lymphoma; NOS, not otherwise specified; PCNSL, primary central nervous system large B‐cell lymphoma; WHO‐HAEM5, 5th edition of the WHO Classification of Hematolymphoid Tumours.

Aggressive B‐cell lymphomas represent a histologic and genetic spectrum of disease entities. B‐cell lymphomas are currently classified based on histologic, genetic, and clinical features according to two closely related classification systems: the World Health Organization (WHO)‐Classification of Hematolymphoid Tumours (WHO‐HAEM5) and the International Consensus Classification (ICC).^1^, ^2^, ^3^, ^4^ While there are some minor differences between these classification systems, they are conceptually similar and represent a foundation for the development of more refined classification taxonomies that take advantage of recent insights into the biology and genetics of aggressive B‐cell lymphoma (Figure 1). Given the rapidly evolving literature dissecting the molecular, genetic, transcriptomic, and epigenetic pathogenesis of DLBCL, a key question is how much molecular information should be captured for newly diagnosed patients with aggressive B‐cell lymphoma inside or outside clinical trials today. Here, we summarize our viewpoint on these questions for diffuse large B‐cell lymphoma (DLBCL) and high‐grade B‐cell lymphoma (HGBL).

## Current status and challenges of gene expression‐based classifications of DLBCL and HGBL

### Cell‐of‐origin (COO) classification of DLBCL

In 2000, the first molecular classification of DLBCL was introduced based on gene expression profiling (GEP) comparing DLBCL patient samples to putative normal B‐cell counterparts.^5^ While some DLBCL samples demonstrated similarities to germinal centre B‐cells, others were more similar to *in vitro* activated B‐cells. According to this inferred COO classification, DLBCL is divided into germinal centre B‐cell–like (GCB), activated B‐cell–like (ABC), or unclassifiable cases. In subsequent studies, patients with GCB‐type DLBCL had a more favourable outcome when treated with R‐CHOP than those with ABC‐type DLBCL.^6^, ^7^

The 4th revised WHO classification from 2016 was the first to incorporate the COO classification, and mandated the recognition of COO subtypes in DLBCL not otherwise specified (NOS) due to their different molecular and biological features, clinical behaviour, and potential therapeutic relevance.^8^ In the 5th revision of the WHO classification, COO subtyping continued to be recommended but no longer mandated, as it was deemed to have limited clinical impact outside of clinical trials.^1^, ^2^

The implementation of GEP‐based COO subtyping in clinical routine was hampered by requirements for RNA extraction from fresh or fresh‐frozen tissue. To overcome such technical hurdles, several alternative classification methods based on immunohistochemistry (IHC) were developed. The most widely accepted one is the Hans algorithm, which uses CD10, BCL6, and MUM1 status by IHC to distinguish GCB and non‐GCB type DLBCLs; the latter being enriched in transcriptionally defined ABC‐type DLBCLs.^9^ Several other methods using different antibodies and algorithms have been developed and are commonly used in clinical practice.^10^, ^11^, ^12^ Despite IHC‐based classifiers being the most widely applied to define the COO subtypes in clinical routine, it is recognized that they imperfectly replicate the transcriptional classification, with misclassification rates ranging from 20% to 50%.^11^, ^13^, ^14^, ^15^, ^16^ This is a key limitation, since the emergence of novel therapeutic agents with COO subtype‐selective activity will necessitate reliable and biologically accurate determination of COO subtypes.

Technological advances have enabled GEP using RNA from formalin‐fixed, paraffin‐embedded (FFPE) tissues. Whole‐transcriptome profiling by RNA‐sequencing (RNA‐Seq) is the most comprehensive GEP method, but can be challenging to perform in clinical laboratories. The Lymph2Cx assay was developed based upon a refined 20‐gene expression COO signature utilizing the nCounter platform,^17^ and showed significant concordance with whole‐transcriptome‐ and IHC‐defined COO subtypes.^18^, ^19^ The Lymph2Cx assay has been used in several clinical trials and has demonstrated an excellent interlaboratory concordance as well as a short turnaround time.^20^, ^21^, ^22^ Other FFPE‐based targeted‐gene expression assays were used in several clinical trials and real‐world studies and showed equally promising results.^23^, ^24^ Such assays therefore allow for cost‐effective, reproducible, rapid, and accurate COO testing and enable COO‐based treatment strategies in DLBCL. An important limitation of such focused GEP assays, especially in the context of clinical trials, is that the information generated is restricted to the COO subtype. In contrast, whole‐transcriptome profiling (RNA‐Seq) allows the application of alternative transcriptional classifiers, including those (discussed below) that incorporate knowledge of the tumour microenvironment.

### RNA‐based detection of molecular high‐grade and dark zone lymphoma

High‐grade B‐cell lymphoma with MYC and BCL2 and/or BCL6 rearrangements (clinically often referred to as double hit lymphoma [DHIT] or triple hit lymphoma) and HGBL, NOS, were newly introduced in the 4th revised WHO classification. Since then, several studies have shown a distinct biology for cases with MYC and BCL2 rearrangement (HGBL‐MYC/BCL2), which usually occur in GCB‐DLBCLs. Diseases with concurrent MYC and BCL6 rearrangement (HGBL‐MYC/BCL6) are biologically heterogeneous and not uniformly attributable to a single COO subtype.^25^, ^26^ Despite the limited molecular and clinical data on this less frequent subset, retrospective analyses so far suggest that HGBL‐MYC/BLCL6, unlike HGBL‐MYC/BCL2, do not exhibit an inferior outcome.^27^ As a consequence, both WHO‐5th and ICC recognize lymphomas with HGBL‐MYC/BCL2 as a distinct entity. In contrast, HGBL‐MYC/BCL6 was removed as a separate entity in the current WHO‐5th classification, and cases are now included as a subtype of DLBCL, NOS, or HGBL, NOS depending on morphologic criteria. The ICC, however, retains lymphomas with HGBL‐MYC/BCL6 as a distinct provisional entity. Recent data highlighted that the molecular architecture of the translocation in HGBCL‐MYC/BCL2 and HGBCL‐MYC/BCL6 are distinct, supporting the notion of different disease entities.^28^ While not reflected in official classifications yet, there is evidence that the negative prognostic impact of HGBL with BCL2 and MYC translocations (HGBL‐MYC/BCL2) is confined to cases where MYC is juxtaposed to an immunoglobulin (IG) partner.^27^, ^29^

Detailed studies on the transcriptional profile of HGBL‐MYC/BCL2 revealed a gene expression signature (DHITSig) that distinguishes HGBL‐MYC/BCL2 from other GCB‐DLBCL.^30^ The 104 genes included in this DHIT‐signature mostly reflected centroblast biology, representing the dark zone (DZ) of the germinal centre (GC) and with low expression of centrocytes or light‐zone (LZ) genes. This observation is the basis for a refinement of the COO‐system based on normal GC B‐cell maturation stages. In parallel, a “molecular high grade” (MGH) signature with a Burkitt‐like gene expression profile was identified by a different group.^31^ Both DHITSig and MHG identified almost all cases of HGBL‐MYC/BCL2, along with similarly sized populations in which MYC/BCL2 rearrangements were not identified by flourescent *in situ* hybridization (FISH). Subsequent sequencing analysis revealed that many of these cases carried cryptic rearrangements at these gene loci.^32^ Upon observation that the “DHIT‐signature” is also exhibited by Burkitt lymphomas, it has been renamed the “Dark Zone signature (DZ signature)”, and has been widely applied in clinical trials and observational studies.^33^, ^34^ The DZ signature has also been incorporated in a concise gene expression panel using the nCounter platform (DLBCL90), which can stratify both GEP‐based COO subtypes and the DZ signatures.

### RNA‐based signatures capturing the tumour microenvironment

The availability of clinical and molecular annotated cohorts and the advancement in deconvolution technologies allowed the transcriptional characterization of the malignant cells and the tumour microenvironment (TME) simultaneously with the same assay. By leveraging cell type‐specific gene expression signature matrices, deconvolution methods allow inferring cell composition and expression profiles of individual cell types of a given tumour sample from bulk gene expression profiling. A prominent example of this technology is CIBERSORT, which works on fresh, frozen, and FFPE tissue samples.^35^, ^36^ In recent years, several groups have developed similar technologies and algorithms and applied them to lymphoma cohorts in order to define different TME subtypes/ecosystems.^37^, ^38^, ^39^ The common conclusion is that TME signatures capture a crucial extra element of lymphoma biology that extends beyond COO subtypes. Such ecotypes/TME subtypes may therefore constitute a meaningful biomarker to inform novel treatments that may be of particular relevance to therapies that exploit the antitumor immune response. However, the limited overlap between studies suggests that TME profiling is not yet ready for routine clinical application. Future studies leveraging single cell and spatial profiling are likely to unravel previously unappreciated cell types and cell states, and will likely lead to further refinement of RNA‐based classification systems.^40^, ^41^, ^42^ An important consideration is that these refined and newly emergent signatures can all be applied retrospectively, and correlated with clinical outcomes in clinical trial cohorts that have captured whole‐transcriptome (RNA‐Seq) data.

### Actionability of RNA‐based signatures

Although the COO has informed our understanding of DLBCL biology, the ABC/GCB distinction does not currently influence treatment selection in DLBCL. As such, in most centres, formal COO distinction by gene expression profiling is rarely performed. Emerging data from *post‐hoc* analysis of the POLARIX trial suggest that the added value of polatuzumab vedotin may be preferentially seen in ABC cases.^22^ If confirmed in prospective trials, these findings might provide a greater imperative for formal COO testing in routine clinical practice. They also demonstrate the critical importance of including comprehensive molecular profiling in prospective clinical trials to identify subtype‐specific drug activity that may not have been predicted in advance. Importantly, if treatment decisions are contingent on COO subtypes, it is essential to base these decisions on robust gene expression assays rather than IHC‐based proxies.

Similarly, in a recent Phase 1b/2 study evaluating the combination of venetoclax, ibrutinib, prednisone, obinutuzumab, and lenalidomide (ViPOR) in relapsed or refractory DLBCL, higher response rates have been observed in non‐GCB cases as well as in high‐grade B‐cell lymphoma with MYC and BCL2 rearrangement.^43^ Another example for how RNA‐based classifications might ultimately guide treatment decisions is the REMoDL‐B trial.^44^ This study randomized patients to receive R‐CHOP +/− the proteasome inhibitor bortezomib. Although no benefit was seen in DLBCL as a whole, missing the primary endpoint of the trial,^44^ improved progression‐free survival (PFS) and overall survival (OS) was seen in the ABC‐type DLBCL in the final analysis of the trial.^24^ This finding fits with our biological understanding of bortezomib targeting nuclear factor kappa‐B (NF‐KB) activation in ABC‐type DLBCLs. Unexpectedly, a benefit was also observed in molecular high‐grade (MHG) lymphomas.^24^ The importance of the aforementioned novel transcriptional classes, which also take the TME into account, was highlighted in a reanalysis of the REMoDL‐B trial. When the study was reanalyzed with respect to DLBCL ecotypes, the LE5 subtype, characterized by TME interactions resembling those of healthy lymphoid tissue, predicted responsiveness to bortezomib.^39^ This represents another example where subtype‐specific responses are not always predictable from our current understanding of DLBCL biology, reinforcing the value of comprehensive molecular profiling in prospective clinical trials. REMoDL‐B illustrates how when this broad molecular data are made available to the research community, it acts as a valuable and enduring research resource to evaluate the impact of emerging molecular signatures and disease subtypes upon treatment response. Such observations of subtype‐specific responses to therapy allow us to form hypotheses that need to be tested in future prospective trials.

## Genetic classifications

### Current approaches for comprehensive genetic classification of DLBCL

**Figure 2:**
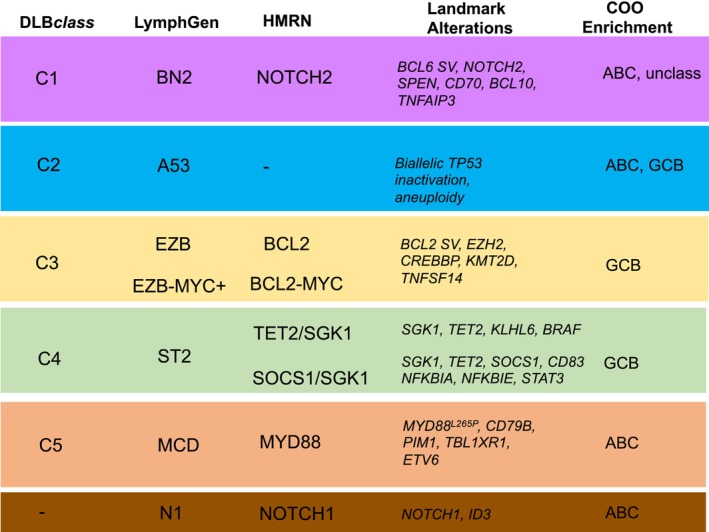
Overview of genetic classes, landmark alterations, and associated cell‐of‐origin subtypes. ABC, activated B‐cell DLBCL subtype; GCB, germinal centre B‐cell DLBCL subtype.

In 2018, two large exome sequencing studies discovered that DLBCL can be subdivided into groups of patients that share similar repertoires of genetic alterations.^45^, ^46^ This gave rise to the LymphGen and DLB*class* taxonomies. Each DLBCL subtype appeared to exploit different oncogenic pathways, suggesting they represent distinct biological entities. There was considerable overlap between the two initial studies. This overlap increased further in a reanalysis of the NCI group.^47^ Further validation was provided by an independent study (HMRN) that applied a broad targeted sequencing panel to 928 cases of DLBCL and reached similar conclusions regarding the genetic substructure of DLBCL.^48^ The DLBCL subtypes and associated key elements of the three genetic studies are summarized in Figure 2.

It was notable that some of the DLBCL subtypes shared genetic features already recognized in the context of other clinically/pathologically classified groups of lymphoma. The MCD/C5 cluster was characterized by mutations that activate the B‐cell receptor and NF‐*κ*B pathways. Almost all cases were associated with an ABC transcriptional signature and the mutation profile overlapped strongly with that reported in extranodal lymphomas such as breast, testis, and primary central nervous system (CNS) lymphomas.^47^, ^49^ EZB/C3 cases were enriched for rearrangement of *BCL2* and mutations in chromatin modifier genes such as *EZH2*, *CREBBP*, and *KMT2D*. These cases were strongly associated with a GCB transcriptional subtype and shared its genetic signature with those found in follicular lymphoma. The BN2/C1 DLBCLs were enriched in ABC and transcriptionally unclassified cases and exhibited frequent *BCL6* chromosomal translocations and activating mutation of *NOTCH2*. This genetic profile resembles that seen in Marginal zone lymphoma, although evidence that BN2/C1 cases represent direct transformation from indolent MZL has not been reported. ST2/C4 clustered cases are almost all GCB in origin. They are strongly enriched for mutations of *SGK1*, *TET2*, *SOCS1*, and genes related to the JAK/STAT and ERK pathways. Their genetic profile resembles that seen in nodular lymphocyte predominant Hodgkin lymphomas. The A53/C2 cluster was characterized by widespread aneuploidy and biallelic inactivation of *TP53*. Finally, a small *NOTCH1*‐mutated cluster (N1) was exclusively defined in the LymphGen classification.

This level of consensus between the main genetic subtyping studies is considerable, and suggests that the concept of distinct genetic subtypes of DLBCL is both real and reproducible. However, differences do exist between the classification systems, most notably the proportion of cases that are classified, ranging from almost every patient in the DLB*class* study, 73% in the HMRN study, and 54% in the LymphGen studies. A second important difference between classifications is the ability to classify an individual case. Each classification was generated by clustering of a large, pooled cohort of patients. At present the LymphGen classifier is available to the community to prospectively classify an individual case. The DLB*class* classifier has been developed and will very soon be available.

### Comprehensive genetic subtyping and prognosis

Genetic classification aims to capture biological differences between subgroups of DLBCL. A separate question is whether biological subtypes are associated with prognostic differences in patients treated with conventional immunochemotherapy. There is general agreement across studies that ST2/C4 cases are associated with an excellent prognosis, while MCD/C5, N1, and EZB‐MYC cases display an inferior prognosis.^45^, ^46^, ^48^, ^50^ However, caution must be exercised in interpreting the impact upon prognosis due to inherent bias associated with retrospective studies performed using archival tissue and low numbers of patients analysed in certain subgroups not balanced for clinical risk factors. The historical example of double‐hit lymphoma shows how our beliefs regarding the prognosis of DHIT have evolved considerably over the last decade from the dismal prognosis reported from early retrospective studies^51^ to a more modest reduction in outcome.^27^, ^33^ The true impact upon prognosis of genetic classification will not become clear until genetic classification becomes more widely applied in standard practice and larger real‐world analyses are performed. An important message is that prognostication is not the primary reason to perform genetic classification—rather, it is to identify biologically discrete groups of patients that may respond differently to biologically targeted therapies.

### Comprehensive genetic subtyping guiding precision therapy

At the time of publication, we do not believe that we are yet in a position to use molecular subtypes to guide therapy in routine clinical practice. This should be based upon robust evidence from future prospective and randomized clinical trials. However, it is notable that, when molecular profiling has been applied to in trials, evidence of subtype‐specific responses has emerged.

One example is the PHOENIX trial,^23^ which randomized 838 patients with DLBCL to R‐CHOP +/− ibrutinib. When considering all cases, there was no benefit to the addition of ibrutinib. However, when a nonstatistically powered, *post‐hoc* analysis was restricted to younger patients with the MCD genetic subtype, the 3‐year PFS improved from 43% to 100%.^16^ This fits with our biological understanding of MCD DLBCL, which is known to depend upon chronic active B‐cell receptor signal transmitted via BTK, the target of ibrutinib. A similar survival benefit was also seen in N1 DLBCL. This was not necessarily predictable from biology, emphasizing how we are not yet able to predict optimal therapy. An important caveat of this study is the small number of patients for whom genetic profiling data were available, with the above observations based upon only 11 ibrutinib‐treated MCD patients. The excess toxicity associated with ibrutinib in elderly patients, in whom the MCD subtype is more common, highlights that besides better classification we also need better compounds. Currently, trials are ongoing to readdress the targetability of ABC/MCD‐enriched cases with next‐generation novel BTK inhibitors.

The GUIDANCE‐01 trial is an example of a prospective trial in which targeted therapy was tailored as a genetic subtype‐specific addition to the R‐CHOP backbone.^52^ Genetic profiling using a small mutation panel and FISH was performed during cycle 1 of R‐CHOP. Subtype‐directed therapy was added to R‐CHOP from cycle 2 onwards. Pooled results across all subtypes showed improved PFS and OS. While the definition of these surrogate subtypes, and the experimental therapies selected, have proven debatable, this study demonstrates the feasibility of real‐time subtype assignment and provides proof‐of‐concept for how subtype‐specific therapies might be tested. A much larger follow‐up trial (GUIDANCE‐02) is ongoing.

It is worth noting that in many clinical trials the information required to associate molecular characteristics, including genetic subtypes, with clinical outcomes is either not acquired or not released. The lack of freely available, comprehensive molecular profiling in aggressive B‐cell lymphoma studies, even in landmark trials, represents a missed opportunity to advance our understanding and hinders progress in developing biologically targeted therapies for DLBCL.

## Outlook of molecular classifications in aggressive B‐cell lymphoma

### Role of molecular classifiers for molecular agnostic therapies

One argument against the inclusion of comprehensive but costly molecular profiling in clinical trials is that almost every successful new therapy for DLBCL in recent years acts in a way that is presumed to be agnostic to the molecular subtype, instead targeting features that are common to all B‐cell lymphomas. Examples include antibody drug conjugates such as polatuzumab vedotin, CAR‐T therapy, and bispecific antibody therapy. However, as discussed earlier, molecular determinants of response start to emerge once the appropriate analysis is performed. In the context of polatuzumab vedotin, there is a suggestion from *post‐hoc* and subsequent pooled analyses that the added benefit of polatuzumab vedotin may preferentially be seen in ABC transcriptional subtypes.^53^ It is surprising that, despite the widespread approval of polatuzumab vedotin in many countries, we do not yet have a definitive answer to this question. This supports our assertion that comprehensive molecular profiling should be a core component of every prospective clinical trial in DLBCL, even in cases where the therapy is assumed to be agnostic to the molecular subtype. Importantly, these data should then be made available to the research community.

Emerging data are starting to reveal molecular determinants of the response to CAR‐T therapy.^54^ These molecular features do not necessarily map to currently recognized genetic subtypes, emphasizing the need for broad molecular profiling to establish the molecular features of sensitivity and resistance to novel therapies. Laboratory‐based research has recently identified protein glycosylation as a major determinant of the response to polatuzumab.^55^ The glycosylation pathway is subject to frequent genetic alteration in DLBCL; however, genes in this pathway do not contribute to the above‐discussed genetic subtypes and are rarely included on targeted sequencing panels. As such, it is currently impossible to assess whether these compelling laboratory data maintain predictive power in patients. This illustrates an important caveat; that trial‐associated molecular profiling should not become over‐focused on small panels designed to infer a given genetic subtype or state. We recommend that profiling in clinical trials should be as comprehensive as possible, ideally including whole‐transcriptome and either whole‐exome or broad sequencing panels. Once available to the research community, these data would provide the crucial opportunity to integrate clinical trial outcomes with new biological data emerging from laboratory‐based discovery research.

### Liquid biopsies for genotyping and minimal residual disease monitoring

While profiling tumour bulk remains standard practice, noninvasive liquid biopsy assays have recently emerged as a viable alternative to tissue‐based diagnostics. These assays utilize DNA isolated from either circulating tumour cells (CTCs) or circulating, cell‐free DNA (cfDNA) from blood plasma to identify the properties of malignant cells. Advantages and disadvantages compared to tissue‐based sequencing are summarized in Table 1. In aggressive B‐cell lymphomas, liquid biopsies have been applied in multiple clinical contexts, which can be mainly subdivided into genotyping and minimal residual disease detection.

**Table 1:** Advantages and disadvantages of tumour sequencing and liquid biopsies

Method	Advantages	Disadvantages
Tumour sequencing	Broad availability	Limited material availability, especially in the case of core needle biopsies
	Relatively cost‐effective	Measurement of only a portion of the tumour, leading to limited representation of the entire tumour
	Numerous tools available for analysis with a high degree of standardization	Fixation artefacts in FFPE material
Liquid biopsy	Minimally invasive, allowing for serial sampling	Lack of standardization
	Better representation of tumour heterogeneity (“spatial heterogeneity”)	High costs due to deep sequencing required for low allele frequency (“needle in a haystack”)
	Can be performed on tumours in locations difficult to biopsy (e.g., brainstem)	Complex bioinformatics

FFPE, formalin‐fixed paraffin‐embedded.

Various studies have demonstrated the utility of ctDNA profiling for genotyping and classification purposes, with a high concordance between plasma and tissue profiling in detecting mutations and other genetic alterations.^56^, ^57^ Plasma may therefore provide an easily accessible source of tumour DNA for genetic profiling, overcoming problems frequently associated with limited biopsy material. This approach can be leveraged not only for genetic subtyping but also to distinguish between solid and lymphoid cancers, which is particularly useful in diseases that are difficult to biopsy, such as CNS lymphomas.^58^, ^59^, ^60^ Beyond genetic profiling, liquid biopsies also allow for the inference of transcriptional activity of malignant cells and the TME. This technology has shown potential for disease detection and classification in aggressive B‐cell lymphoma and classic Hodgkin lymphoma,^61^, ^62^ with the limitation that the genotyping and classification capability deteriorates in cases with very low levels of ctDNA.^56^

The ctDNA levels have been shown to largely reflect tumour burden, correlating closely with clinical risk factors such as stage and the international prognostic index (IPI).^63^, ^64^, ^65^ High baseline ctDNA levels are associated with poorer treatment outcomes and are independent of currently applied clinical stratifiers.^63^, ^64^, ^65^ Moreover, ctDNA has merit as a dynamic risk factor. A rapid drop in ctDNA and achieving ctDNA negativity is associated with favourable treatment outcomes in both frontline and relapsed/refractory disease.^54^, ^63^, ^65^, ^66^, ^67^, ^68^, ^69^, ^70^ Currently, the best way to assess ctDNA and its independence of next‐generation imaging technologies is an area of active clinical investigation. While it is unclear which is the best assay for ultra‐sensitive minimal residual disease (MRD) detection and which assay will pave its way to approval by the authorities, it is clear that ctDNA‐based MRD will become a standard end‐of‐treatment response assessment in clinical trials and routine care in the not too distant future.

### Barriers to implementing molecular profiling

There is currently no clear consensus on the type and scale of molecular profiling required in routine practice and clinical trial settings. As a consequence, often only minimal molecular data are captured. Although it is possible to apply a simplified version of the above genetic classifications using a small, focused panel of 10–20 genes, we caution against this for the reasons discussed and urge that clinical trials should include molecular profiling as comprehensively as possible—ideally whole‐transcriptome and either whole‐exome or at least a broad targeted panel that allows recapitulating each of the current genetic subtypes. We argue that this molecular profiling should be a core component of every clinical trial, and that provision of molecular profiling, freely available at the time of trial publication, should be considered a marker of good clinical trial practice in the eyes of regulators and publishing journals.

It is important to consider the barriers to implementing such a recommendation. Costs associated with performing comprehensive sequencing are considerable and frequently cited as a barrier to inclusion of molecular profiling in clinical studies. However, the costs associated with a lost learning opportunity are also considerable. The blanket use of expensive, targeted drugs where benefit may be restricted to a subset incurs a significant and long‐term cost to healthcare funders, without benefit to patients. It is therefore our opinion that molecular studies should be considered an essential component of any clinical trial and that academic or industry sponsors should have a responsibility to fund this profiling and to ensure data are made freely available at the time of publication. Outside of clinical trials, any future approval of subtype‐specific therapies should act as a commercial incentive for pharma to support routine molecular profiling in routine diagnostic laboratories, or to provide appropriate companion diagnostic tests to accompany use of their product.

Important technical barriers include the significant variation in variant calling and driver annotation pipelines. Moreover, there is variation in which variants should be considered clinically significant, and how best to report these variants. We lack consensus on balancing the benefit versus the cost of requiring a germline control, or even precisely what this control should be. This means that different laboratories may differ in the exact genetic profile they report for an identical tumour. As sequencing technology becomes more widely applied it will be essential to develop harmonized reporting standards and appropriate programs to ensure quality control.

A cause for hesitancy when considering the genetic classification of lymphoma is that our current taxonomy may not be the ultimate answer. Indeed, it is highly likely that further evolution of these classifiers will occur. This may result from an improved understanding of the impact of alterations in the noncoding genome, or novel insights into the contribution of the host immune system and microenvironment to the biology of the lymphoma. New technologies that profile the epigenome—i.e. modifications to DNA and histones regulating gene expression such as methylation marks—or function at the single‐cell or spatial level, will likely refine our understanding of DLBCL biology and impact future subtyping. However, the level of consensus around current transcriptional and genetic subtypes suggests that these will continue to form the foundation for DLBCL subclassification for the foreseeable future.

The source of tissue to sequence remains a problem. Lymphoma biopsies are almost always placed into formalin, which increases the noise of sequencing. The increasing use of core needle biopsies means available tissue is limited, and frequently exhausted during the diagnostic process. The rapid development of plasma DNA sequencing technologies may provide a much more easily accessible source of tumour DNA for genetic profiling. These technical and logistical problems will not be solved in academic trials. They will require us to develop real‐world experience and to develop harmonized approaches to molecular profiling that are robust across different laboratories.

### Recommendation for molecular profiling to be implemented in clinical trials

**Figure 3:**
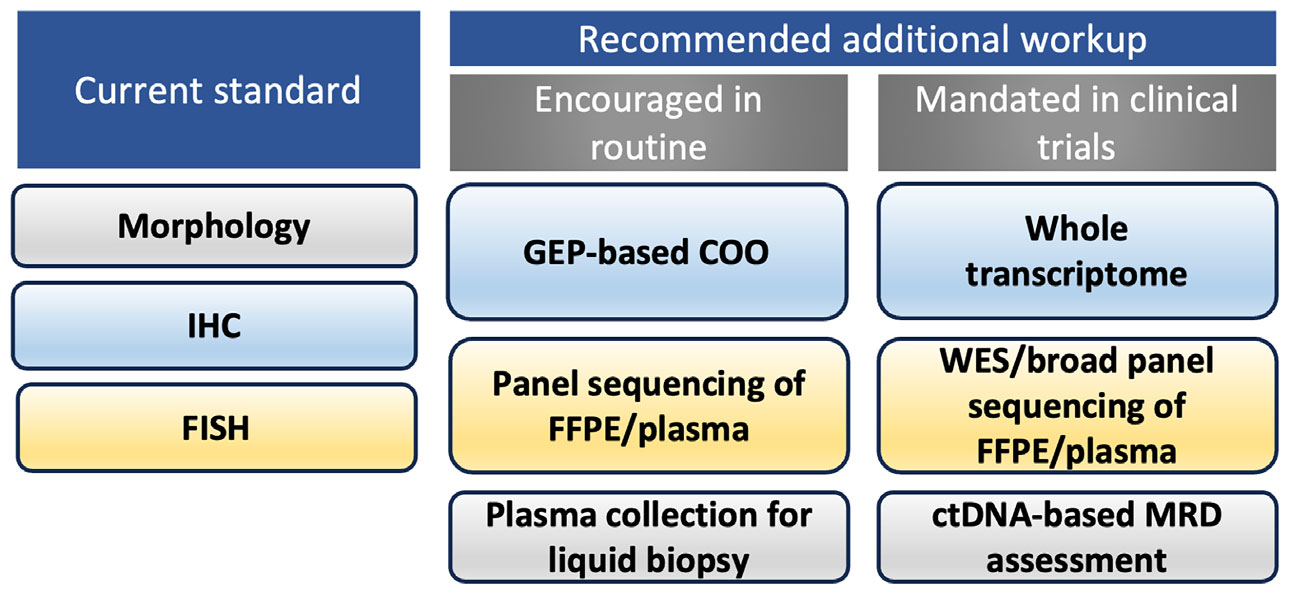
Recommended workup of aggressive lymphoma. IHC, immunohistochemistry; FISH, fluorescence *in‐situ* hybridization; GEP, gene‐expression profiling; COO, cell‐of‐origin; FFPE, formalin‐fixed paraffin‐embedded; WES, whole‐exome sequencing; ctDNA, circulating tumour DNA; MRD, minimal residual disease.

We recommend that every prospective clinical trial in DLBCL/HGBL should be accompanied by comprehensive molecular profiling (Figure 3). This should include whole‐transcriptome profiling and preferentially also include whole‐exome sequencing from FFPE and/or plasma derived DNA with germline control, applied to all enrolled patients. An inferior but more cost‐effective alternative would be the use of a broad targeted panel for DNA sequencing. Collection of plasma and assessment of ctDNA‐based MRD is strongly encouraged. These investigations should be considered a core component of the trial and should be made publicly available at the time of trial publication. Doing so would allow the application of current transcriptional and molecular subtypes, including the RNA‐based TME signatures. It would allow the identification of subtype‐specific responses, including those not anticipated from our preclinical understanding. These findings would generate hypotheses to fuel future prospective trials of subtype‐specific responses. Broad molecular profiling would also future‐proof trials against evolution of molecular classification, equipping them with the data required to evaluate the treatment‐specific impact of emergent molecular classifiers. Finally, it provides trials with the common molecular currency needed to interface with findings that emerge from laboratory studies of the molecular pathogenesis of lymphoma and studies into the molecular determinants of response and resistance to specific targeted therapies.

### Recommendation for molecular profiling to be implemented in routine clinical practice

The situation in routine clinical practice is different, since molecular results do not currently inform decision‐making. Moreover, many routine diagnostic services lack the required infrastructure, tissue handling pathways, and technical expertise required to perform molecular profiling. These logistical and technical hurdles will require real‐world solutions that may vary from site to site. By building real‐world experience of genetic profiling in lymphoma we will overcome these challenges and start to accrue knowledge banks required to exploit the full potential for subtype‐directed therapy to improve outcomes in DLBCL. Essentially, we propose that unless we begin to build the experience and technical expertise today, it will be extremely difficult to implement such approaches tomorrow. To this end, we recommend that, if possible, RNA‐based COO detection, targeted sequencing using a broad lymphoma panel, and collection of plasma should be performed in all centres where there is infrastructure and the resources to do so (Figure 3). We recognize that these recommendations impose a substantial financial burden and may not be feasible in all countries and healthcare systems. In countries or healthcare systems where resources are limited and treatment options cannot exceed R‐CHOP, a practical approach to management of DLBCL may not require any form of molecular profiling. In contrast, large academic centres in first‐world countries should have a responsibility to lead in building the knowledge, expertise, and infrastructure to enable implementation of molecularly targeted therapy in DLBCL.

## Conclusion

As outlined in this article, our understanding of the molecular underpinnings driving aggressive B‐cell lymphoma biology has radically evolved over the past 25 years. We now stand at the brink of a new era in lymphoma treatment, transitioning from a ‘one‐size‐fits‐all’ approach to truly personalized therapies. Although advanced molecular insights and data may not yet impact therapeutic decision‐making, there is an urgent need to better incorporate molecular methodology into clinical trials and ultimately into routine clinical practice. This will generate the necessary evidence to shift biomarkers from being merely descriptive to becoming critical contributors in guiding optimal treatment strategies for these diseases. The promise of personalized medicine in aggressive lymphomas now needs to be realized.

## Author contributions

All authors contributed equally to the design, writing, and approval of the article.

## Conflict of interest

SKA: consultancy for foresight diagnostics; BC: Inventor on patent applications related to DLBclass; DE: research funding from Eisai, Chugai, Nipponshinyaku; KD: consultancy for Astra Zeneca, Abbvie, ADC Therapeutics, Beigene, Bristol Myer Squibb, Amgen, Genentech, Genmab, Pharmacyclics, Incyte, ONO Pharmaceuticals, Celectar. Research Funding from Genentech, ONO Pharmaceuticals, Merck, Kymera. DJH: research funding from GSK and Astra Zeneca.

## Data Availability

Data sharing not applicable to this article as no datasets were generated or analysed during the current study.
